# Experience of the Evolving Roles of Nurses in Genetic Counselling in Spain: An Interpretive Description Study

**DOI:** 10.1111/inr.70137

**Published:** 2025-11-25

**Authors:** Daniel García‐Gutierrez, Cristina García‐Salido, Estel la Ramírez‐Baraldes

**Affiliations:** ^1^ Departamento de Enfermería, Facultad de Ciencias de la Salud de Manresa Universitat de Vic‐ Universitat Central de Catalunya (UVic‐UCC) Barcelona España; ^2^ Grupo de Investigación en Simulación e Innovación Transformativa (GRIST) Instituto de Investigación e Innovación en Ciencias de la Vida y de la Salud de la Cataluña Central (Iris‐CC) Vic España; ^3^ Institut de Recerca i Innovació en Ciències de la Vida i de la Salut a la Catalunya Central (IRIS‐CC) Vic Spain; ^4^ Unitat de Cures Intensives Althaia Xarxa Assistencial Universitària de Manresa Manresa Spain

**Keywords:** applied qualitative research, genetic counselling, interpretive description, interprofessional collaboration, nursing, professional practice, Spain

## Abstract

**Aim:**

This study explores the experiences and challenges faced by Spanish nurses involved in genetic counselling.

**Background:**

Genetic counselling is a vital component of modern healthcare, yet its regulation and professional recognition vary globally. In Spain, despite growing demand, nurses specializing in genetics lack formal recognition, limiting both patient care quality and professional development.

**Introduction:**

Nurses play a key role in delivering genetic counselling through patient education, emotional support, and care coordination. However, their role remains structurally undefined in Spain, highlighting the need for policy and educational reforms.

**Methods:**

We conducted an interpretive description study grounded in an interpretivist–constructivist stance. Data were generated through semi‐structured individual interviews, one focus group, and field notes. Analysis followed the interpretive description analytic logic (iterative coding, constant comparative analysis, integrative synthesis) to inductively develop practice‐relevant interpretive themes.

**Results:**

Four subthemes emerged: professionalism, experience, needs, and team collaboration. Nurses emphasized the value of continuous training, therapeutic relationships, and interdisciplinary teamwork. Structural challenges included limited resources, lack of formal recognition, and insufficient supervision.

**Discussion:**

Nurses are essential in providing patient‐centred genetic counselling, yet face barriers such as inadequate training, institutional support, and unclear professional boundaries. Emotional intelligence and teamwork were highlighted as key to effective practice.

**Conclusion:**

This study calls for urgent reforms to enhance training, recognition, and integration of nurses into genetic counselling services in Spain.

**Implications for Nursing and Health Policy:**

This research underscores the importance of recognizing nursing as a valuable and viable option for expanding genetic counselling services. Compared with other healthcare professionals, nurses possess unique strengths such as strong communication skills, long‐term patient engagement, and holistic care approaches, essential for effective genetic counselling. Their proximity to patients and ability to provide continuity of care make them ideal candidates for this evolving role. By prioritizing nursing‐led genetic counselling, Spain can improve access to services while offering new professional opportunities for nurses. Institutional support, formal recognition, and accredited postgraduate education pathways are needed to ensure high‐quality service delivery and career development. Investing in nursing within clinical genetics not only enhances patient outcomes but also strengthens the sustainability and efficiency of genomic healthcare in Spain.

## 1 Introduction

Genetic counselling emerges as an essential component in contemporary medical care, addressing the complex clinical and psychological ramifications of genetic diseases (Bambini et al. 2009). This integrative process is based on the meticulous interpretation of family and personal medical history in conjunction with a solid understanding of the principles of genetic inheritance (Majstorović et al. 2021). Through education on the genetic implications of diseases and available testing options, genetic counselling empowers individuals and families to make informed decisions and effectively adapt to the risks and challenges they face (Anderson et al. 2015; Skirton 2017).

Internationally, the situation and regulation of genetic counselling vary considerably. In countries like the United States, Canada, Japan, Australia, and the United Kingdom, genetic counselling is an established profession with accrediting bodies and specific training programmes. In Europe, the European Board of Medical Genetics (EBMG) was created to establish professional standards in medical genetics and genetic counselling with the aim of improving patient care and safety (Abacan et al. 2019). It arose in response to the lack of cohesion among European countries regarding educational standards and the need to establish competencies for genetics professionals.

The global state of genetic counselling as a profession in 2018 revealed the presence of approximately 7000 genetic counsellors distributed in around 28 countries (EBMG 2013). In many countries, genetic counselling began with nurses or other health professionals who received training from clinical geneticists. In Spain, although in 2018 there were around 70 genetic counsellors and a master's programme in Barcelona, the lack of formal recognition makes it difficult to obtain employment in this field. Unlike other countries that have ‘genetics nurses’, Spain has not yet fully developed this option, creating a gap in specialized genetic care.

The EBMG (Blondeaux et al. 2023) recommends that both genetic counsellors and genetics‐specialized nurses be trained at the master's level with specific degrees in genetic counselling and genetics nursing, respectively. The curriculum of the Master's in Genetic Counselling in Europe covers a wide range of topics, from counselling and communication skills to knowledge in human and medical genetics, ethics, and practical preparation for professional practice. These contents ensure that students gain a deep understanding and the necessary skills to offer quality genetic counselling, respect human rights, maintain confidentiality, and provide compassionate and non‐directive support to clients and their families.

Despite the growing demand from both professionals and patients, the regulation of the genetic counsellor profession in Spain is limited. This lack of organization and regulation not only affects the quality of care provided to the affected population but also results in unnecessary healthcare expenditure. The lack of standardization in genetic services and the training of professionals in the field highlight the urgent need to establish policies and regulations that support the proper development of genetic counselling in the country.

Nursing plays a fundamental role in the field of genetic counselling (Anderson et al. 2015). Nurses are key in delivering genetic counselling to patients, providing information and support both before and after testing. They can effectively and compassionately communicate results, help interpret information, and offer emotional support to patients and their families, which is crucial for managing stress and anxiety associated with genetic testing (Skirton 2017). Nurses can also educate patients about the risks and benefits of genetic tests and the implications of the results, helping them make informed decisions about their medical care. Additionally, they can coordinate care among different health professionals, ensuring comprehensive and patient‐centred care (Barr et al. 2018).

An integrative review of previous literature (Kirk et al. 2011) investigates the current practice of genetic counselling by nurses amid the debate on whether this should be a specialized role or part of general nursing practice. It concludes a lack of consensus on the scope and manner in which nurses provide this type of counselling and recommends further research on accreditation, role recognition, support, and education for nurses in this field, highlighting their potential to significantly contribute to supporting individuals affected by genetic issues due to their wide availability (Skirton et al. 2007; Calzone et al. 2013; Álvaro‐Sánchez et al. 2021).

Although the participation of nurses in genetic counselling has been recognized internationally, in Spain, there is still little regulation and formal recognition of this professional role. The available literature shows a significant gap in terms of qualitative understanding of the lived experiences of nurses involved in this field.

Specifically, in Spain, a previous review (Calzone et al. 2018) about genetic services highlights the lack of recognition of geneticists and genetic counsellors as healthcare professionals, which hinders access to these services with low regulation and formal recognition of this professional role. This underscores the lack of consensus on the extent and way nurses provide genetic counselling, highlighting the need for further research in this area. However, there is a lack of evidence on the specific experience of nurses in this area, and the available literature shows a significant gap in terms of qualitative understanding of the lived experiences of nurses involved in this field. This study arises precisely from that need: to explore for the first time qualitatively the specific experiences and challenges of Spanish nurses in the context of genetic counselling, in an environment where their role lacks formal structure and institutional recognition. Therefore, the main research question, ‘What are the specific experiences and challenges faced by Spanish nurses involved in genetic counselling?’, takes on special relevance as it addresses an underexplored area with important practical and educational implications. This research not only provides novel empirical knowledge but also offers a solid basis for future initiatives aimed at professional development, continuing education, and effective integration of nurses in clinical genetics services in Spain (Kirk et al. 2014).

The main objective of this study is to delve into the experiences of Spanish nurses who perform genetic counselling and specifically identify the challenges they face in their practice and to fill a gap in the existing literature to provide valuable information with the aim of improving training, support and recognition of nurses who play a crucial role in the field of genetic counselling (Skirton et al. 2007; Sandelowski 2000; Thorne 2016).

## 2 Methods

### 2.1 Study Design

This study is positioned within an interpretivist–constructivist paradigm and adopts interpretive description (ID) as the overarching methodological approach. ID is an applied qualitative methodology developed within the health disciplines to generate clinically useful, practice‐informed understandings of experiential phenomena, moving beyond surface description to an interpretive account that can inform care, education, and policy (Thorne et al. 1997; Thorne et al. 2004; Maykut and Morehouse 1999). We selected ID because our question sought to explain how nurses understand and enact their evolving roles in genetic counselling and to produce insights directly translatable to service design, training, and professional recognition in Spain.

Distinguishing ID from alternative approaches. In contrast to qualitative description (Sandelowski 2000, 2010), which prioritizes a low‐inference descriptive summary, ID explicitly pursues a coherent interpretive synthesis with disciplinary relevance. Phenomenological designs were not pursued because our analytic intent was across‐case pattern explanation to inform practice rather than idiographic explication of lived experience.

For novice researchers or clinicians, this approach can be understood as one that values the unique voices and lived experiences of participants, recognising that reality is not fixed but is constructed through social interactions and personal meaning. In practical terms, it means interpreting how nurses understand and engage emotionally with their professional roles, which is essential when studying under‐explored or poorly defined healthcare roles, such as genetic counselling in Spain.

An interpretative design was chosen to explore the context and nurses' experiences in genetic counselling aspects that cannot be fully captured using quantitative methods, enabling the interpretation of underlying meanings and identification of relevant patterns for professional practice (Van Manen 2003; Braun and Clarke 2021; Braun and Clarke 2013).

### 2.2 Data Collection

Data collection was conducted through semi‐structured interviews with a focus group and field notes, aiming to obtain detailed information about participants' personal and professional experiences. Individual interviews provided a more intimate space to explore deeper aspects with an average duration of 60 minutes, while the focus group encouraged interaction among nurses, generating an enriching dynamic that added new perspectives derived from group exchange lasting 90 minutes. In both cases, all information was audio‐recorded for subsequent transcription and data analysis.

Interviews followed a structured guide specifically developed for this study based on research objectives and validated by experts in qualitative methodology and clinical genetics. The guide covered key topics such as perception of professional role, received training, daily challenges, therapeutic relationship with patients, and interdisciplinary collaboration. Some included questions were: ‘How would you describe your experience providing genetic counselling?’ ‘What are the main challenges you face in your work?’ or ‘How has your genetics education developed?’ These questions guided conversations, although flexibility was maintained to follow emerging lines during each dialogue, ensuring both structure and openness in topic exploration.

The focus group used an adapted guide, encouraging group discussion, promoting contrast between viewpoints. Initial rules were established to ensure respectful communication, balanced participation, and confidentiality. The group was moderated by one of the researchers, supported by a second moderator, while a third team member took notes and recorded relevant observations regarding nonverbal language, expressed emotions, and observed communication patterns. This triangulation in data collection (individual interviews + focus group + field notes) enhanced the richness and depth of the subsequent analysis.

Inclusion and exclusion criteria were clearly defined at the beginning of the study. Only nurses who provided some types of genetic counselling were included, whether as part of a multidisciplinary team or independently, with a minimum professional experience of 10 years in nursing and at least two years as genetic counsellors.

In the Spanish context, all participants were registered nurses (*enfermeras tituladas*) with official university degrees in nursing, accredited by the Spanish Ministry of Education, and legally authorized to practice. Their qualifications reflect the standard professional training for nursing in Spain, which includes a four‐year bachelor's degree (*Grado en Enfermería*). This ensured that all participants met national standards for clinical competence, providing a consistent professional baseline for the study.

Specific training in genetics was not required to capture a broad view of current practical realities in Spain. Other types of healthcare professionals whose work was not directly linked to genetic counselling or who were not actively part of services dedicated to this area were excluded. The sample was obtained through convenience sampling, a common strategy in qualitative studies where access to qualified participants may be limited due to the specificity of the topic and the scarcity of specialized personnel in Spain. Data collection concluded once data saturation was achieved, understood as the point where no new subthemes or relevant perspectives emerged, ensuring adequate depth without unnecessary redundancy.

The research study was conducted from December 2023 to May 2024 and with a sample size of eight nurses from the Spanish territory meeting, aged 30–50 years, and the inclusion and exclusion criteria previously outlined. This sample size is consistent with qualitative studies using reflexive thematic analysis aimed at experiential, pattern‐based accounts, privileging information richness over statistical representativeness. As mentioned in recognized methodological literature, the optimal number of participants in qualitative studies ranges between six and twelve, depending on the study objective and the complexity of explored topics. In this case, the collected data offered rich informative density sufficient to identify consistent thematic patterns and reach solid conclusions.

### 2.3 Data Analysis

Data analysis was conducted through iterative cycles of familiarization, inductive coding, constant comparative analysis within and across cases, memo‐writing, matrixing, and integrative synthesis to develop interpretive themes that illuminate clinically salient patterns. Team dialogues and reflexive notes documented analytic decisions and evolving interpretations. We sought explanatory coherence and practice relevance, checking emergent interpretations against disconfirming data and interrogating fit with the disciplinary context (Thorne et al. 1997). Representative extracts are presented to enhance interpretive authority while retaining participant confidentiality. (Kalpokaite and Radivojevic 2019; Gadamer 1998; Maykut and Morehouse 1999; Van Manen 2003; Flick 2018). We attended to established ID quality criteria: epistemological integrity (alignment between question, methods, and interpretivist stance), representative credibility (adequate sampling for variation; thick, representative exemplars), analytic logic (transparent audit trail of coding, comparisons, and integrative moves), interpretive authority (reflexive memos, team debriefings, and member‐oriented checks on interpretive summaries), moral defensibility and disciplinary relevance (ethical conduct; salience to nursing in genetic counselling), and pragmatic obligation (actionable implications for education, service design, and policy) (Thorne et al. 1997). We did not employ codebook/coding‐reliability procedures aimed at consensus metrics; rather, analysis followed ID's flexible, reflexive logic to support conceptual insight applicable to nursing practice.

This combination of analytical techniques, along with a justified sampling strategy and transparency in procedure description, reinforces the methodological quality of the study. However, some limitations inherent to its qualitative nature are acknowledged, such as narrative subjectivity, limited generalizability, and absence of longitudinal follow‐up. Nevertheless, the chosen approach proved suitable for addressing the stated objective, providing a deep, rich, and relevant insight to inform future training policies, professional recognition, and improvements in genetic counselling service provision within the Spanish context.

Finally, it should be noted that the design of the study was carried out by the three authors, as well as in participation in the collection of information (individual interviews, focus groups, or field diaries). On the other hand, the data analysis was a task performed by the first and second authors and with participation in the drafting and revision of the article by the three authors.

### 2.4 Ethical Consideration

This study was approved by the Ethics Committee of UVic‐UCC University (approval no. 141/2021) on 24 January 2021. All participants were contacted in advance and, before starting any activity, were asked to sign the written informed consent form on paper and were provided with a copy of it and all the information about the study.

## 3 Results

### 3.1 Characteristics of the Results

As analysis progressed, we generated initial codes across transcripts and iteratively constructed, reviewed, and defined candidate themes. The interpretive thematic structure comprised four interpretive subthemes—professionalism, experience, needs, and collaboration—each with several subthemes (see Table 1) (Flick 2018). The results are displayed using a concrete nomenclature in order to preserve the confidentiality of the participants: ‘P’ followed by their identification number corresponds to each participant, ‘EI’ indicates individual interview, and ‘GF’ refers to the focus group.

**TABLE 1:** Interpretive themes and subthemes.

Themes	Subthemes
Professional development	Professionalism
	Experience
Resource management	Needs
	Collaboration and team dynamics

*Source*: Own elaboration.

### 3.2 Main Interpretive Themes and Subthemes

As analysis progressed, we generated initial codes and iteratively constructed candidate themes. Through reviewing and refining, we defined a coherent set of themes and subthemes that captured patterned meaning across participants. The interpretive thematic structure comprised four subthemes, professionalism, experience, needs, and collaboration (see Table 1) (Resta et al. 2006; Skirton et al. 2010). The information was progressively articulated into four core subthemes: professionalism, experience, needs, and team collaboration and dynamics (see Table 2). These interpretive subthemes reflect essential aspects related to professional practice within the context of genetic counselling and provide an integrative framework for understanding the experiences and challenges faced by nurses in this specific setting.

**TABLE 2:** Definition of the research interpretive subthemes.

Subthemes	Definition
Professionalism	Encompasses aspects related to ethical conduct, responsibilities, and skills necessary in professional practice. Includes the ability to work effectively in a team, proper distribution of responsibilities, maintenance of clear professional standards, and continuous training and development to improve clinical practice.
Experience	Addresses the diversity of work and educational experiences of health professionals, as well as the variety of clinical approaches and practices used in different contexts. Also considers the evolution of roles and responsibilities over time in response to changes in practice and healthcare demands.
Needs	Refers to the obstacles and conditions affecting the effective delivery of health services and the quality of care. Includes organizational restrictions and limited resources that may impact the ability of health professionals to provide adequate care, as well as work pressures and additional challenges in the work environment. Also addresses ethical and confidentiality dilemmas that may arise in clinical practice.
Collaboration and Team Dynamics	Refers to the process by which professionals work together in a coordinated manner, sharing responsibilities, knowledge, and resources to achieve common goals. This dynamic involves establishing strong interdisciplinary relationships, promoting effective communication, fostering an environment of trust and mutual respect, and recognizing and leveraging the individual strengths of each team member. It also involves understanding and managing the hierarchical structure within the team to ensure equitable role distribution and efficient decision‐making.

*Source*: Own elaboration.

This analytical process concluded with the identification of two main themes associated with the professional development and resource management (see Table 3). These interpretive themes emerged through subthemes (Gadamer 1998), allowing the findings to be integrated and synthesized coherently and contextually. The data illustrate how participating nurses describe their professional roles from multiple perspectives, ranging from continuing education and professional development to therapeutic relationships with patients, institutional resource needs, and collaborative work within multidisciplinary teams. Each of these aspects was explored in depth through individual interviews, which provided an intimate space to address complex personal issues, while the focus group encouraged peer exchange, generating new shared interpretations and perspectives that enriched the overall understanding of the studied phenomenon.

**TABLE 3:** Definition of the research interpretive themes.

Themes	Definition
Professional development	Addresses the promotion of continuous professional development. Includes participation in training and continuous education programmes, as well as the acquisition of interpersonal skills, leadership abilities, and collaboration among health team members to ensure comprehensive and coordinated patient care.
Resource management	Addresses the challenges and limitations faced by professionals in their work environment. Includes the management of limited resources and organizational restrictions, and the demands of the healthcare system. Also refers to ethical challenges and effective collaboration among team members.

*Source*: Own elaboration.

## 4 Discussion

The analysis of the data revealed several significant findings related to the practice of genetic counselling from the perspective of nurses in Spain. In this section, the results obtained through a comprehensive analysis of the individual interviews, the focus group, and the field notes collected during the study are presented. Each subtheme is broken down into specific categories that provide a detailed understanding of the nurses' experiences. The results are discussed using the following notation to preserve the confidentiality of the participants: ‘P’ followed by their identification number corresponds to each participant, ‘EI’ indicates individual interview, and ‘GF’ refers to the focus group.

### 4.1 Interpretive Subtheme: Professionalism

In the ‘Professionalism’ interpretive subthemes, it stands out as a fundamental pillar in the practice of nurses, encompassing training and professional development as well as the ability to establish strong therapeutic relationships. It was observed that participants emphasized the importance of continuous training and professional development as essential elements to maintain high standards of practice in genetic counselling because nurses must stay updated on advances in genetics and medical technology.

Participants emphasized the importance of continuous training and professional development as key to staying current in the fast‐evolving field of genetic counselling. One nurse explained, ‘I believe that continuous training and professional development are very important to stay updated in a constantly evolving field like genetic counselling’ (EI_P6). The need for educational support was also linked to the initial challenges some professionals faced when entering this area. As another participant noted, ‘The transition to this new role was difficult, but with perseverance, I was able to adapt and grow professionally’ (GF_P2). Participants also advocated for the inclusion of genetics‐related content in undergraduate nursing curricula, recognizing that early academic exposure is essential. As expressed in the focus group, ‘We highlight the need for training in genetics for nurses and its integration into undergraduate education, emphasizing the importance of continuous education’ (GF_P1).

The study highlights the importance of professionalism and continuous development in the field of genetic counselling, a need reflected in the voices of the participating nurses. The quotes highlight the relevance of continuous training and professional growth to stay updated in a constantly evolving field like genetics (Tortajada Lohaces et al. 2019). This aligns with the standards established for genetics‐specialized nurses and genetic counsellors in Europe (Luis et al. 2019), where the need for specific education and key competencies to offer a specialized service to clients or patients is recognized. Continuous training not only allows professionals to stay up to date with advances in genetics but also helps them provide quality care and establish strong therapeutic relationships, as highlighted in the professional and educational standards document (EBMG 2010, 2013). According to participants, nurses undergo a progressive process of growth and skill acquisition in genetic counselling. While initial feelings of uncertainty are common, these tend to diminish as experience is gained, highlighting the central role of practical exposure in professional development. These findings demonstrate the importance of practical experience and continuous learning in acquiring clinical competencies and improving the quality of care provided.

The participants highlighted the critical role of communication skills and the therapeutic relationship in the practice of genetic counselling. They emphasized that explaining complex genetic information in a way that is accessible to patients is not straightforward. As one nurse put it, ‘Communicating complex concepts in an understandable way for patients is an art’ (EI_P1). This communicative competence was seen as foundational to effective counselling, with the same participant adding, ‘Establishing an effective therapeutic relationship is fundamental to the success of treatment’. Similarly, empathy and active listening were identified as essential pillars in building trust with patients, with another nurse affirming, ‘Empathy and active listening are key pillars in the therapeutic relationship with patients’ (EI_P3).

Beyond the technical aspects, participants also stressed the emotional and psychological demands of the role. One participant underlined the need for specific skills in delivering bad news and offering emotional support, noting that these require both practical experience and self‐driven learning: ‘I emphasize the importance of having skills in communicating bad news and providing emotional support to patients, highlighting the importance of practical experience and self‐training in this aspect’ (GF_P5). Another focus group participant reflected on the evolution of their role from traditional nursing to one that mirrors the responsibilities of a counsellor: ‘I feel very much like a counsellor because, of course, we don't do nursing as such now, but… we try to transmit knowledge to patients by empathizing, listening, etc.’ (GF_P7).

These reflections reveal how the therapeutic relationship is not only a technical skill but also a deeply interpersonal process, shaped by empathy, emotional resilience, and the ability to connect with patients in meaningful ways. They emphasize the need to communicate complex concepts in an understandable way for patients, implying a clear and effective connection with them. Participants noted that a strong therapeutic relationship is fundamental to the success of counselling, suggesting that its quality directly influences the outcomes of this. They also emphasize empathy and active listening as key elements in this relationship. These perceptions corroborate the ‘Professional and Educational Standards for Genetic Counsellors in Europe’ (Setiawan et al. 2021) and the ‘Code of Professional Practice for Genetic Counsellors in Europe’ (Jenkins et al. 2001), which affirm that counsellors play a fundamental role in identifying and addressing individual or family needs while highlighting their ability to provide genetic counselling empathetically and client‐centred, significantly contributing to promoting the well‐being of patients.

These findings are consistent with previous research (Berninger et al. 2021) that affirms that the nurse plays a fundamental role in the provision of genetic counselling services. Their role ranges from facilitating the counselling process to educating patients about the nature of their genetic condition, available treatment options, and long‐term health implications. Additionally, the nurse evaluates the psychosocial status of patients, providing emotional support and ensuring they receive comprehensive and continuous care (Siskind and Atzinger, 2019).

### 4.2 Interpretive Subtheme: Experience

Regarding the ‘Experience’ interpretive subtheme, the importance of continuous learning and constant updating in the field of genetic counselling is highlighted. As mentioned, accumulated experience provides a solid foundation in the field of genetic counselling. However, the challenges of staying up to date with constant advances in genetics are also highlighted. This aspect underscores the importance of continuously staying informed, asking questions, and actively seeking new evidence and knowledge.

Participants reflected on how professional experience plays a pivotal role in navigating the complexities of genetic counselling. Several nurses described the steep learning curve they faced when entering the field, marked by initial uncertainty and a sense of being unprepared. One participant explained, ‘My incorporation into the genetic counselling team arose due to a vacancy and my previous experience in colon screening…. I felt lost at first, but over time I acquired the necessary skills for my professional development’ (EI_P8). Similarly, another admitted, ‘It was a bit difficult at first, I told them that I didn't know what I was getting into, and I thought I wouldn't make it, but over time…. I enjoyed it a lot’ (GF_P7).

The learning process was described not only as technical but also as deeply personal and continuous. Nurses acknowledged that genetic knowledge evolves rapidly, and staying up to date requires sustained effort. As one participant noted, ‘Genetics is constantly changing…. It's a challenge because the spectrum of alterations is very large…. It is a continuous process of learning and improvement’ (GF_P6). This sentiment was echoed by another nurse who shared their ongoing struggle with uncertainty in variant interpretation: ‘What was once considered a variant of uncertain significance is now nothing…. That's where the struggle is, and where you have to stay informed every day… with your desire to learn’ (EI_P4).

These experiences underscore the dynamic nature of the field and the importance of lifelong learning. Participants portrayed their journey not as a linear path but as one shaped by perseverance, adaptability, and intrinsic motivation to grow professionally within a constantly changing scientific landscape.

This underscores the need for specific training programmes that provide nurses with the necessary skills to develop professionally in the field of genetic counselling. These results align with previous research (Lea and Lawson, 2000; Eggert 2017) in the United States and other countries that mention the importance of building a dynamic and effective educational infrastructure that increases the number of genetic counsellors graduated from accredited training programmes. This implies the implementation of specific training programmes for health professionals, such as nurses who wish to specialize in genetic counselling.

In terms of professional development, nurses demonstrate a commitment to continuous learning and the acquisition of interpersonal skills to ensure comprehensive and coordinated patient care (Maykut and Morehouse 1999; Van Manen 2003). However, they face challenges in managing limited resources and the demands of the healthcare system, highlighting the need for additional support and resources to optimize the delivery of genetic counselling services (De Jesús and Mitchel 2017).

### 4.3 Interpretive Subthemes: Needs

Participants expressed a variety of structural challenges that affect their ability to provide optimal genetic counselling, highlighting a general lack of resources, unclear professional pathways, and insufficient recognition of their role. Many nurses reported difficulties when entering the role, especially regarding the absence of clearly defined protocols or expectations. One participant explained, ‘When I started, they told me they were going to open an agenda for me, but many things here were not applicable… everything else has been about finding my own way and opening my mind… but naming and structuring them was difficult for me’ (EI_P4). This reflection illustrates the lack of formal guidance and the need for greater institutional support in defining and structuring nursing roles within genetic services.

The shortage of resources was another commonly reported barrier. One nurse shared, ‘I found a lack of resources at the beginning… it was a constant obstacle, but I learned to make the most of what we had to provide the best possible care’ (EI_P6). Despite their adaptability, participants consistently underscored the need for adequate tools and support systems to ensure high‐quality service delivery.

In addition to operational constraints, there was a strong call for professional recognition and formalization of genetics nursing as a distinct area of practice. One focus group participant highlighted, ‘We discussed the absence of a recognized specialty in genetics for nurses and the need for more structured training and adequate recognition of our work in this field’ (GF_P3). Others pointed to the legal ambiguity surrounding responsibilities, especially in clinical documentation: ‘We discussed the signing of genetic counselling reports by a doctor, addressing legal and responsibility aspects…. There is a lack of formal recognition of the role and structural limitations faced’ (GF_P5).

Together, these insights reveal a pressing need for structural reforms, clearer role definitions, and formal training programmes that support the evolving responsibilities of nurses in genetic counselling. Without these, their capacity to provide consistent and comprehensive care remains compromised.

The challenges in structuring the work are evident in the initial difficulty of establishing and properly naming nursing agendas, suggesting a lack of established protocols and resources to guide their work. Adapting to a new environment may require additional effort and an open mind to overcome these difficulties. Additionally, the shortage of resources emerges as a recurring obstacle to providing the best possible care. This lack of resources can limit professionals' ability to address the complex needs of patients and their families, highlighting the critical importance of having adequate resources to ensure the quality of care provided.

Furthermore, the urgent need for recognition and training in different fields is highlighted. The lack of formal recognition of specialties and the need for more structured training underscore the importance of continuous training and professional recognition to ensure excellence in care. Lastly, structural and legal limitations represent another set of challenges faced by health professionals in their daily practice. Addressing legal and responsibility issues in clinical practice is crucial to improving the effectiveness and quality of care provided to patients. Overcoming these structural barriers is essential to ensuring a conducive work environment and quality care.

In the needs category, a clear demand for a type of guidance or supervision similar to that received by other professionals has also been revealed. Genetics nurses express the need for structured support to improve their practice and offer more comprehensive care to their patients and families.

Several participants emphasized the emotional toll that genetic counselling can have on nurses, particularly when supporting families during difficult diagnoses. One nurse shared that ‘sometimes we have very tough consultations… patients and stories that have marked me due to the emotional impact of the diagnosis on the family because many times the patient feels guilty for being a carrier of that disease’ (EI_P6). These emotionally charged situations led many to express the need for more formalized support. As one participant explained, ‘I think that as genetics nurses, we could benefit from a type of guidance or supervision like the one received by other professionals in our field…. It would help us a lot to deal with the complexities we face day to day… especially when dealing with emotional and psychosocial aspects of genetic conditions in families’ (GF_P2). Another participant echoed this sentiment, noting, ‘I feel that we lack that more structured support that other colleagues have. A kind of mentoring or supervision would help us improve our practice and provide better care to our patients and their families’ (GF_P3).

This finding aligns with the highlighted importance of supervision in genetic counselling as established by the EBMG (Jenkins et al. 2001). That is, genetic counsellors operating in clinical settings require both clinical and counselling supervision, as they must explore the emotional and psychosocial ramifications of genetic conditions. Therefore, empathic engagement, genuine interaction, and management of transfer dynamics become indispensable. Thus, counselling supervision serves several purposes: providing psychological support, fostering self‐awareness, exploring responses to patients, managing emotional burden, facilitating professional reflection, and mitigating burnout risks (Majstorović et al. 2021; Corno Caparrós et al. 2018; MacFarlane et al. 2016; Jenkins et al. 2001).

### 4.4 Interpretive Subthemes: Collaboration and Team Dynamics

Finally, the ‘Collaboration and Team Dynamics’ interpretive subtheme highlights the importance of communication and interdisciplinary collaboration in the context of genetic counselling and patient care.

The importance of interdisciplinary collaboration and effective team dynamics emerged as a central theme in the participants’ accounts. Nurses described how their roles have expanded over time, gaining autonomy and becoming central figures in patient care within genetic counselling teams. One participant shared, ‘Now many times, all these questions are directed to us… they send me something about a man, such and such, don't you think we should do…? Or do you think we should have asked someone else?’ (EI_P1). This illustrates the growing trust and responsibility placed on nurses by their colleagues.

Teamwork was consistently described as essential for delivering comprehensive and high‐quality care. As one nurse explained, ‘In our genetic counselling team, we recognize the value of teamwork as a fundamental element to provide comprehensive and quality genetic counselling’ (GF_P1). This was reinforced by another participant who noted the gradual shift in perception of nurses' roles, stating, ‘With the years we have been existing, even silently, everyone is now starting to pick up this knowledge a bit… we are beginning to enjoy this trust from other professionals’ (GF_P5).

Support from the wider multidisciplinary team was also framed as a necessary condition for effective practice. One nurse emphasized, ‘The support of the multidisciplinary team is essential to offer comprehensive and effective care to patients’ (GF_P8). These reflections suggest that collaborative environments not only enhance care delivery but also contribute to the recognition and consolidation of nurses’ roles in genetic counselling services.

It is observed how nurses assume broader and more autonomous roles in this process, suggesting a decentralization of responsibilities. Additionally, the essential collaboration between different specialties to address the complex demands of patients is highlighted, integrating consultations and working as a team to provide a comprehensive approach to care. The value of teamwork is recognized as fundamental to providing quality genetic counselling. Over time, experience has strengthened trust and collaboration among professionals, facilitating more effective care. These observations about the importance of the therapeutic relationship are supported by recent research (MacFarlane et al. 2016), such as the study on the effects of anxiety on the experience of supervised clinical rotations of novice genetic counselling students. This study found that common themes included the balance between support and guidance from supervisors and the importance of feedback. Our findings offer a practice‐relevant explanation of how nurses navigate and make sense of genetic counselling roles under conditions of limited recognition and resources, thereby identifying concrete leverage points for education, supervision, and service organization.

### 4.5 Limitations

Despite the valuable findings obtained, it is important to acknowledge several potential limitations of this project. First, the sample size was relatively small, with only eight participants. Although data saturation was achieved, the limited number may affect the generalizability of the results. A larger and more diverse sample could have provided a broader and more representative perspective of nurses’ experiences in genetic counselling.

Second, the use of convenience sampling may have introduced selection bias. Nurses who agreed to participate might differ in relevant ways—such as having greater interest or more positive attitudes towards genetic counselling—compared with those who declined to participate. These differences could compromise the validity of the results.

Third, as is typical in qualitative research, findings are inherently subjective and based on self‐reported perceptions. While steps such as triangulation and participant feedback on interpretive summaries were employed to enhance credibility, the data reflect individual experiences rather than objective metrics.

Fourth, the study did not include a longitudinal follow‐up, which limits insight into how nurses’ perceptions and experiences might evolve over time in response to changes in policy, education, or institutional support.

Finally, the focus on Spanish nurses provides both a limitation and a strength. While it restricts the generalization of the findings to other geographical or cultural contexts—given that experiences and challenges may vary across countries due to different healthcare systems and policies—it also addresses a previously underexplored area. The study contributes valuable insights to the Spanish context and may serve as a basis for international comparisons, enriching the global understanding of the evolving role of nurses in genetic counselling. As an interpretive qualitative account situated in a specific national context, our aim was explanatory insight for practice, not statistical generalization. Interpretations are therefore context‐bound yet transferable to similar settings where disciplinary structures are comparable.

## 5 Conclusions

This study provides valuable insights into the evolving role of Spanish nurses in genetic counselling, shedding light on their experiences, challenges, and contributions to clinical practice. The findings reveal that nurses play a critical role in delivering comprehensive, patient‐centred genetic counselling, particularly through effective communication, emotional support, and interdisciplinary collaboration.

Key themes include the importance of professionalism, underlined by the need for continuous training, ethical competence, and strong therapeutic relationships with patients and families. Nurses emphasized the necessity of staying updated in a rapidly advancing field and adapting their skills to meet complex clinical demands.

The lack of formal recognition and structured training for genetics‐specialized nursing in Spain emerged as a major challenge. Participants reported difficulties related to limited resources, unclear professional boundaries, and insufficient institutional support. These barriers hinder both the quality of care and the professional development of nurses in this field.

The study highlights the value of interdisciplinary teamwork and shared decision‐making in improving patient outcomes. Nurses described taking on broader, more autonomous roles within multidisciplinary teams, contributing significantly to patient education, psychosocial support, and long‐term care coordination.

Finally, the results underscore the urgent need to develop formal educational programmes, institutional policies, and supervision frameworks tailored to nurses working in genetic counselling. Investing in these areas will enhance service delivery, promote professional growth, and ensure sustainable integration of nursing into genetic healthcare services in Spain.

## 6 Implications for Nursing and Health Policy

The findings of this study highlight significant implications for nursing practice and health policy, particularly regarding the integration of nurses into genetic counselling services in Spain. As the demand for genetic counselling continues to grow due to advancements in genomic medicine and increased patient awareness, it becomes imperative to formally recognize and support the role of nurses within this field, integrating them into national healthcare frameworks.

One of the most pressing issues identified is the need for formal recognition of nursing as a specialized area within genetics. Currently, there is no structured professional pathway or official accreditation system for nurses working in genetic counselling in Spain. This absence of institutional recognition limits career development opportunities, hinders service delivery, and ultimately compromises the quality of care provided to patients and their families. Establishing a recognized specialty or certification in genetic nursing would clarify professional competencies, scope of practice, and career progression, thereby enhancing both the credibility and effectiveness of nursing contributions in clinical genetics.

In addition to professional recognition, there is an urgent need for the implementation of structured educational programmes tailored specifically to nurses. Participants in the study emphasized the lack of formal education in genetics at both undergraduate and postgraduate levels. Incorporating core content on human genetics, genetic counselling, communication strategies, and psychosocial support into basic nursing curricula would ensure that future professionals are adequately prepared. Offering specialized master's degrees or continuing education courses focused on genetic counselling could help bridge existing gaps and align Spain with international standards such as those proposed by the EBMG.

Health policy must also evolve to support institutional and regulatory changes that facilitate the integration of nurses into multidisciplinary genetic services. Policymakers should collaborate with nursing associations and genetic societies to develop clear guidelines, legal frameworks, and ethical standards that define and protect the role of nurses in genetic counselling. This includes ensuring that nurses are authorized to perform and document genetic counselling independently under appropriate supervision and with defined responsibilities. Such measures would not only enhance professional autonomy but also improve access to genetic services for the general population.

Workplace support and supervision structures should be established to address the emotional and psychological demands faced by nurses in this field. Genetic counselling often involves delivering complex and distressing information, which requires strong emotional resilience and interpersonal skills. Providing access to regular supervision, peer support groups, and mental health resources can significantly reduce burnout, improve job satisfaction, and enhance the overall quality of care delivered to patients and their families.

Moreover, promoting interdisciplinary collaboration through policy initiatives is essential. Nurses have demonstrated their capacity to contribute meaningfully to patient education, psychosocial support, and long‐term care coordination in genetic settings. Encouraging collaborative models of care through policy‐level incentives can optimize resource use, improve patient outcomes, and foster innovation in service delivery. Strengthening teamwork between nurses, genetic counsellors, physicians, and other allied health professionals will be key to providing comprehensive, patient‐centred care.

This study underscores the importance of integrating nursing into the field of genetic counselling, especially considering that nurses already possess many of the required competencies, such as advanced communication, empathy, and patient‐centred care, that are essential for effective genetic counselling. Compared with other healthcare professions, nurses are uniquely positioned to provide holistic, long‐term support to individuals and families affected by genetic conditions. Their ability to build therapeutic relationships, interpret complex medical information, and coordinate care across disciplines makes them ideal candidates for expanding roles in genetic services.

Despite these strengths, genetic nursing remains largely unrecognized in Spain, limiting both professional opportunities and the potential to improve patient care. The results of this research call attention to the urgent need for comprehensive reforms in nursing education, professional regulation, and healthcare policy to fully leverage the potential of nurses in genetic counselling. Addressing these challenges through targeted interventions will not only enhance the professional role of nurses but also contribute to building a more resilient, competent, and sustainable workforce capable of meeting the growing demands of genomic healthcare in Spain.

Furthermore, this study serves as a valuable tool for raising awareness among healthcare institutions and nursing universities about the relevance and potential of genetic nursing. It highlights the need to prioritize the incorporation of nurses into genetic counselling roles before other healthcare professionals who may not have the same level of training in patient communication, emotional support, or long‐term care coordination. By investing in nursing‐led genetic counselling services, academic institutions and health centres can promote a more efficient, compassionate, and integrated model of care that benefits both patients and the healthcare system.

## Author Contributions

**Daniel García‐Gutiérrez**: study design, data collection, study supervision, manuscript writing, and critical revisions for important intellectual content. **Cristina Garcia‐Salido**: study design, data collection, data analysis, study supervision, manuscript writing, critical revisions for important intellectual content. **Estel·la Ramírez‐Baraldes**: study design, data collection, manuscript writing, and critical revisions for important intellectual content.

## Funding

The authors have nothing to report.

## Conflicts of Interest

The authors have no conflict of interest in relation to this article.

## Data Availability

Data sharing is not applicable to this article as no datasets were generated or analysed during the current study.
